# A GA-independent, membrane-associated pathway regulates DELLA degradation during carbon/nitrogen stress

**DOI:** 10.1093/plcell/koag037

**Published:** 2026-02-20

**Authors:** Ju-Chen Chia

**Affiliations:** Assistant Features Editor, The Plant Cell, American Society of Plant Biologists; Plant Biology Section, School of Integrative Plant Science, Cornell University, Ithaca, NY, United States

Plants tightly regulate the balance of carbon (C) and nitrogen (N), the 2 most abundant macronutrients required for growth and development. Disruption of this balance triggers characteristic stress responses. For example, excess sugar combined with limited nitrogen creates high C/N ratios (hereafter, C/N stress), leading to reduced photosynthesis, impaired storage lipid breakdown, and increased anthocyanin accumulation in seedlings (Martin et al. 2002). Although the C/N stress response has been well documented, only a limited number of genes underlying this process have been identified. One such component is Arabidopsis Toxicos En Levadura 31 (ATL31), a membrane-associated RING-H2-type ubiquitin E3 ligase that accumulates under C/N stress and promotes seedling growth while reducing stress effects during early development (Sato et al. 2009). On the other hand, the role of the phytohormone gibberellic acid (GA) in abiotic stress responses has been widely studied. Specifically, DELLA proteins are well established as central negative regulators of the GA signaling pathway by repressing GA-responsive gene expression. However, the involvement of GA and its regulators in C/N stress has not been examined.

**Gerardo Carrera-Castaño** and colleagues (Carrera-Castaño et al. 2026) addressed this gap by uncovering a previously unknown link between the GA signaling pathway and C/N stress in Arabidopsis. The authors showed that C/N stress repressed the transcription of the GA biosynthesis gene *GA3ox1*, and reduced GA signaling output in a GA biosensor line, while exogenous GA alleviated C/N stress phenotypes during seedling establishment. Notably, the effect of GA persisted even in genetic backgrounds lacking the ATL E3 ligases ATL31 and ATL6, which have been previously implicated in stress mitigation. These findings indicate that GA and the ATL pathway act independently in regulating C/N stress responses. To identify the specific DELLA proteins involved in the C/N stress response, the authors introduced different combinations of *della* mutants into the *ga1-3* background, in which DELLA proteins hyperaccumulate due to impaired GA biosynthesis. This genetic dissection demonstrated that adaptation to high C/N ratios was shaped by 2 DELLAs, REPRESSOR OF GIBERELLIC ACID (RGA) and GIBERELLIC ACID INSENSITIVE (GAI).

DELLA proteins undergo proteasomal degradation in the nucleus through both GA-dependent and GA-independent pathways when environmental conditions favor growth (Dill et al. 2004; Blanco-Touriñán et al. 2020). These observations, together with previous genetic analyses, raised the possibility that turnover of RGA and GAI contributes to the C/N stress response. Under high C/N conditions, the protein abundance of both DELLAs increased in planta, likely due to reduced GA biosynthesis. Furthermore, yeast 2-hybrid and co-immunoprecipitation analyses showed that both RGA and GAI physically interact with ATL31. Bimolecular fluorescence complementation (BiFC) and Förster Resonance Energy Transfer–Fluorescence Lifetime Imaging Microscopy (FRET-FLIM) assays revealed that these interactions occurred at the plasma membrane, reflecting the membrane-association of ATL31. These interactions promoted cytosolic ubiquitylation and proteasomal degradation of RGA and GAI, suggesting that ATL31 contributes to the C/N stress response by modulating the stability of these DELLA proteins.

Importantly, ATL31-mediated DELLA degradation occurred independently of canonical GA signaling. One line of evidence is that the Arabidopsis lines carrying GA-resistant DELLA variants (Δ17) displayed C/N stress phenotypes comparable to their respective wild types, yet these GA-resistant DELLAs remained sensitive to ATL31-mediated degradation. Moreover, *ATL31* overexpression reduced C/N stress sensitivity, even when GA biosynthesis was compromised. Together, these findings indicate that ATL31-driven DELLA turnover under C/N stress does not require GA. The authors also showed that *ATL31* overexpression under balanced C/N ratios did not affect DELLA-regulated traits during early developmental stages, including seed dormancy, germination, or seedling establishment, highlighting the specific role of the ATL31-DELLA module in high C/N conditions.

In summary, Carrera-Castaño et al. identified new molecular components of the C/N stress response and uncovered a GA-independent, membrane-associated mechanism that modulates the stability of DELLA proteins (see Fig.1) Under C/N stress, reduced GA biosynthesis promotes RGA and GAI accumulation, while the membrane-linked E3 ligase ATL31 interacts with cytosolic RGA and GAI and promotes their ubiquitylation and proteasomal degradation. Together, these 2 pathways fine-tune C/N stress response by altering DELLA protein abundance. Unlike previously established DELLA turnover pathways that operate in the nucleus, the ATL31-DELLA pathway adds another layer of regulation that enables precise adjustment of growth and development under stress.

**Figure 1:**
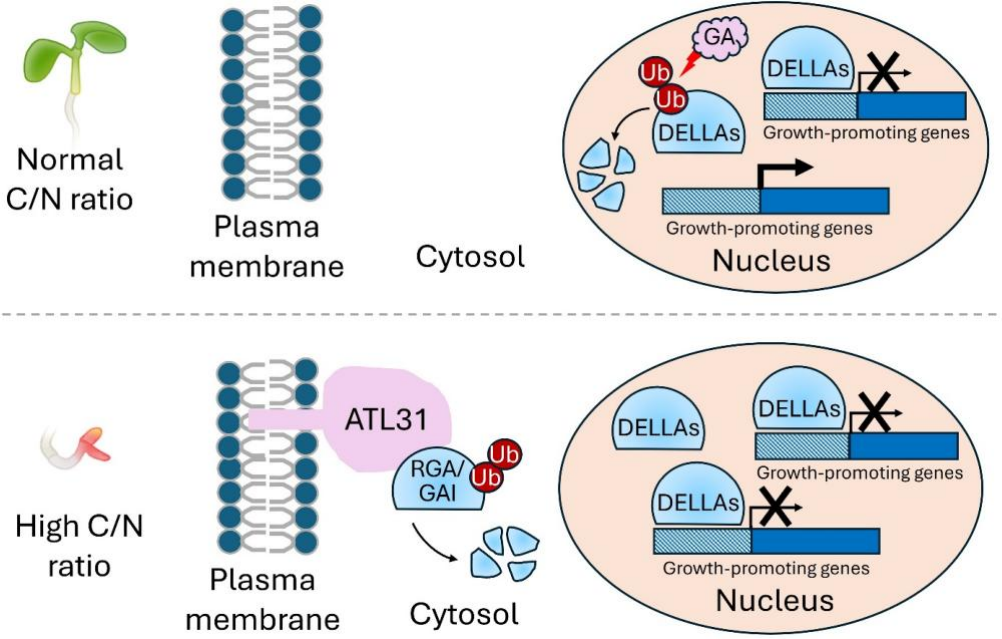
Model of ATL31-mediated DELLA turnover in C/N stress. Under balanced C/N conditions, DELLA proteins are primarily degraded through the canonical GA-dependent pathway in the nucleus to support normal growth. Under C/N stress, reduced GA biosynthesis promotes DELLA accumulation, while membrane-localized ATL31 drives the ubiquitylation and degradation of GAI and RGA, thereby fine-tuning growth responses. Adapted from Carrera-Castaño et al. (2026), Figure 7C.

## Recent related articles in *the plant cell*

Liu et al. (2025) reported that the peptide CmGAST1 promotes flowering in chrysanthemum (*Chrysanthemum morifolium*), with its transcription repressed by a GAI-Calmodulin complex, highlighting a role for GAI in integrating hormonal and developmental signals beyond stress responses.

Bian et al. (2025) characterized transcriptional gene regulatory circuits upstream of NIN-like proteins (NLPs) and their nitrate-responsive gene targets, integrating transcriptional regulation with known post-translational control of NLPs in Arabidopsis and comparing conserved and divergent regulatory wiring in tomato (*Solanum lycopersicum*).

Shanks et al. (2024) reviewed spatiotemporal dynamics of nitrogen sensing and regulatory networks, highlighting how nitrogen signaling is integrated across cell types, organs, and systemic physiological processes.

## Data Availability

No new data were generated or analysed in support of this research.

## Dive Curated Terms

The following phenotypic, genotypic, and functional terms are of significance to the work described in this paper:

ATL6 Gramene: AT3G05200
ATL6 Araport: AT3G05200

ATL31 Gramene: AT5G27420
ATL31 Araport: AT5G27420

GA3ox1 Gramene: At1g15550
GA3ox1 Araport: At1g15550

RGA Gramene: AT2G01570
RGA Araport: AT2G01570

GAI Gramene: AT1G14920
GAI Araport: AT1G14920

## References

[koag037-B1] Bian C et al 2025. Conservation and divergence of regulatory architecture in nitrate-responsive plant gene circuits. Plant Cell. 37:koaf124. 10.1093/plcell/koaf124. 40403157 PMC12205479

[koag037-B2] Blanco-Touriñán N et al 2020. COP1 destabilizes DELLA proteins in *Arabidopsis*. Proc Natl Acad Sci U S A. 117:13792–13799. 10.1073/pnas.1907969117. 32471952 PMC7306988

[koag037-B3] Carrera-Castaño G et al 2026. Membrane-associated DELLA degradation modulates growth under carbon/nitrogen imbalance. Plant Cell. 38:koag013. 10.1093/plcell/koag013. 41578857 PMC13017015

[koag037-B4] Dill A, Thomas SG, Hu J, Steber CM, Sun TP. 2004. The Arabidopsis F-Box protein SLEEPY1 targets gibberellin signaling repressors for gibberellin-induced degradation. Plant Cell. 16:1392–1405. 10.1105/tpc.020958. 15155881 PMC490034

[koag037-B5] Liu W et al 2025. The peptide CmGAST1 integrates calcium and gibberellin signaling to regulate flowering in chrysanthemum. Plant Cell. 37:koaf269. 10.1093/plcell/koaf269. 41219161

[koag037-B6] Martin T, Oswald O, Graham IA. 2002. Arabidopsis seedling growth, storage lipid mobilization, and photosynthetic gene expression are regulated by carbon:nitrogen availability. Plant Physiol. 128:472–481. 10.1104/pp.010475. 11842151 PMC148910

[koag037-B7] Sato T et al 2009. CNI1/ATL31, a RING-type ubiquitin ligase that functions in the carbon/nitrogen response for growth phase transition in Arabidopsis seedlings. Plant J. 60:852–864. 10.1111/j.1365-313X.2009.04006.x. 19702666

[koag037-B8] Shanks CM et al 2024. Nitrogen sensing and regulatory networks: it's about time and space. Plant Cell. 36:1482–1503. 10.1093/plcell/koae038. 38366121 PMC11062454

